# Design of Multivariate Biological Metal–Organic Frameworks: Toward Mimicking Active Sites of Enzymes

**DOI:** 10.1021/acs.inorgchem.4c01988

**Published:** 2024-07-09

**Authors:** Javier Navarro-Alapont, Cristina Negro, Sergio Navalón, Amarajothi Dhakshinamoorthy, Donatella Armentano, Jesús Ferrando-Soria, Emilio Pardo

**Affiliations:** † Instituto de Ciencia Molecular (ICMol), Universidad de Valencia, 46980 Paterna, Valencia, Spain; ‡ Departamento de Química, Universitat Politècnica de València, Camino de Vera s/n, Valencia 46022, Spain; § Dipartimento di Chimica e Tecnologie Chimiche (CTC), Università della Calabria, Rende 87036 Cosenza, Italy

## Abstract

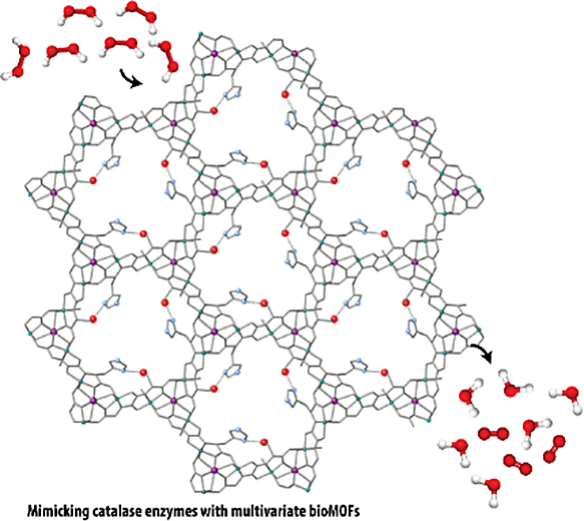

Mimicking enzymatic processes carried out by natural enzymes, which are highly efficient biocatalysts with key roles in living organisms, attracts much interest but constitutes a synthetic challenge. Biological metal–organic frameworks (bioMOFs) are potential candidates to be enzyme catalysis mimics, as they offer the possibility to combine biometals and biomolecules into open-framework porous structures capable of simulating the catalytic pockets of enzymes. In this work, we first study the catalase activity of a previously reported bioMOF, derived from the amino acid *L*-serine, with formula {Ca^II^Cu^II^_6_[(*S*,*S*)-serimox]_3_(OH)_2_(H_2_O)} · 39H_2_O (**1**) (serimox = bis[(S)-serine]oxalyl diamide), which is indeed capable to mimic catalase enzymes, in charge of preventing cell oxidative damage by decomposing, efficiently, hydrogen peroxide to water and oxygen (2H_2_O_2_ → 2 H_2_O + O_2_). With these results in hand, we then prepared a new multivariate bioMOF (MTV-bioMOF) that combines two different types of bioligands derived from *L*-serine and *L*-histidine amino acids with formula Ca^II^Cu^II^_6_[(*S*,*S*)-serimox]_2_[(*S*,*S*)-hismox]_1_(OH)_2_(H_2_O)}·27H_2_O (**2**) (hismox = bis[(S)-histidine]oxalyl diamide ligand). MTV-bioMOF **2** outperforms **1** degrading hydrogen peroxide, confirming the importance of the amino acid residue from the histidine amino acid acting as a nucleophile in the catalase degradation mechanism. Despite displaying a more modest catalytic behavior than other reported MOF composites, in which the catalase enzyme is immobilized inside the MOF, this work represents the first example of a MOF in which an attempt is made to replicate the active center of the catalase enzyme with its constituent elements and is capable of moderate catalytic activity.

## Abstract 2

The present work describes how the fine-tuning of the functionalities decorating the channels of a multivariate biological metal−organic framework (MTV-bioMOF) can constitute a step forward in mimicking the active centers of enzymes.

## Introduction

Metal–organic frameworks^1−4^ (MOFs) are crystalline porous materials that have already not only found a wide diversity of real applications^5^ but also offer exciting promising perspectives in other emerging challenging fields.^6^ Most of these present and potential applications of MOFs arise from the symbiosis between their intrinsic porous nature and outstanding host–guest chemistry.^4,7,8^ For example, recent years have witnessed the appearance of certain pioneering studies reporting the use of MOFs as catalysts replicating/mimicking the activity of natural enzymes, where host–guest chemistry lies at the origin of such properties.^9−16^

There are two main approaches that lead to enzymatic catalysis in MOFs. The first approach consists of the immobilization of the enzyme inside the MOF, which includes insertion within the channels^17,18,27,19−26^ or growing the MOF around the enzyme (biomineralization).^28−36^ However, even if this strategy, a priori, somehow ensures that the integrity of the active site remains unaltered, this is not always the case, as the functional groups decorating the MOFs or the insertion/biomineralization conditions could have a negative influence on them. In addition, it has to be taken into account that the accessibility of the reactants to the cavities where the enzymes are hosted is not always ensured. The second strategy, certainly more challenging, involves attempting to replicate the active enzymatic centers with the functional groups of the ligands and metals forming the MOF.^37−40^ The latter would allow for combining an efficient active center and sufficient space to carry out the enzymatic reaction. However, it is quite evident that efficiently replicating what nature has accomplished over many years of evolution is not a simple task. Therefore, the development of this pathway still requires a great effort from synthetic chemists, but the potential rewards are well worth it.

In this context, the so-called biological MOFs^41,42^ (bioMOFs) – which are prepared by using biofriendly metals and biological ligands (which can be biomolecules or their derivatives) – arise as suitable candidates to achieve replicating active centers of enzymes, and thus, allowing to achieve MOFs exhibiting enzymatic catalysis. This is explained by the fact of combining in the same material the metals and biomolecules (amino acids for instance) that make up the enzymes responsible for this type of catalysis. For instance, among other examples, we have reported recently the hydrolase–like^43^ and glycosidase-like^44^ catalysis by a family of oxamidato-based bioMOFs, derived from different natural amino acids,^43,44,53−56,45−52^ where their catalytic properties arise from the dense decoration of the bioMOFs channels with the functional residues from the constituting amino acids.^43,44^ In addition, a natural evolution and great alternative to “traditional” single-linker MOFs are given by an emerging type of MOFs named multivariate^57^ MOFs (MTV-MOFs or, in this case, MTV-bioMOFs), combining two or more different organic linkers into the same backbone, thus introducing considerable (and sometimes controllable) heterogeneity to a given framework, which offers, with regard to enzymatic catalysis, the possibility of a more effective simulation or mimicking of the active centers of enzymes composed of multiple biomolecules (amino acids).

## Results and Discussion

**Figure 1:**
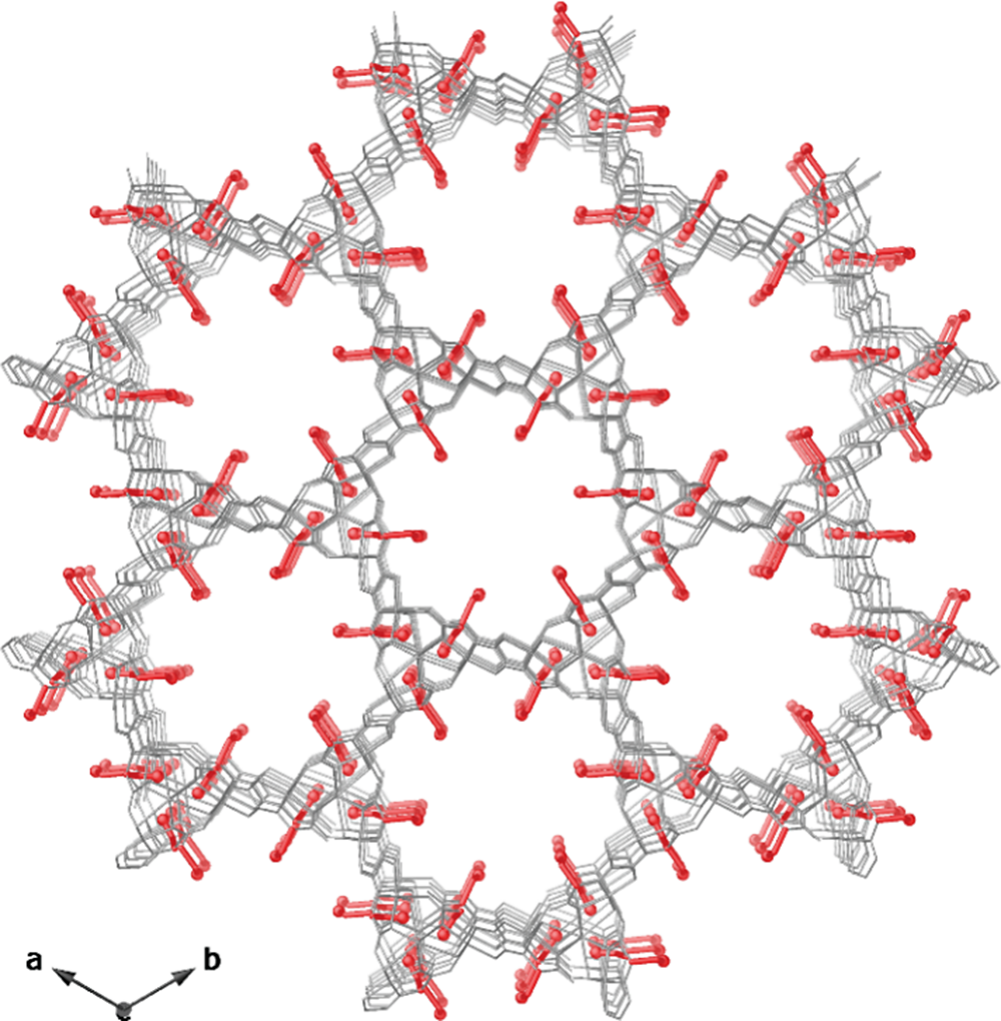
Perspective view of the open-framework of bioMOF **1** along the *c* axis (the crystallization water molecules are omitted for clarity). Copper(II) and calcium(II) ions from the network and organic ligands are depicted as gray sticks for the sake of clarity whereas the amino acid residue from *L*-serine (−CH_2_OH) is represented as red spheres and sticks.

In this context, we have focused our efforts on taking a step forward toward mimicking catalase enzymes with bioMOFs, which play a fundamental role in living cells protecting them from oxidative damage. In particular, we explore here, the catalase-like properties of a previously reported oxamidato-based Cu_6_Ca bioMOF, derived from the *L*-serine amino acid (Figure 1 and Scheme S1), with formula {Ca^II^Cu^II^_6_[(*S*,*S*)-serimox]_3_(OH)_2_(H_2_O)}·39H_2_O^43,56,58^ (**1**) (where serimox = bis[(S)-serine]oxalyl diamide). Moreover, we also report the preparation of a completely novel MTV-bioMOF constructed by combining stoichiometric amounts (2:1) of the amino acids *L*-serine and *L*-histidine with formula {Ca^II^Cu^II^_6_[(*S*,*S*)-serimox]_2_[(*S*,*S*)-hismox]_1_(OH)_2_(H_2_O)}·27H_2_O (**2**) (where hismox^59^ = bis[(*S*)-histidine]oxalyl diamide) (Scheme S1). Remarkably, this ratio of ligands in the final compound is obtained regardless of the proportion of precursor complexes used initially. Thus, even if serimox/hismox ratios of 1:1, or even 1:2, are used, the resulting serimox/hismox ratio is always 2:1 (Tables S2–S5). These results suggest that 33% is the maximum amount of histidine ligands that can be included in the framework, most likely due to steric effects, as a consequence of the bulkier residue of the histidine amino acid. Remarkably, bioMOF **1** exhibits certain catalase-like activity, arising from the copper(II) centers within the framework. In addition, the novel reported MTV-bioMOF **2** outperforms **1**, most likely due to the programmed presence of 33% of histidine residues (−CH_2_C_3_H_3_N_2_) decorating the pores, which are known to play an important role in catalase enzymes^60^ (Scheme S2).

**Figure 2:**
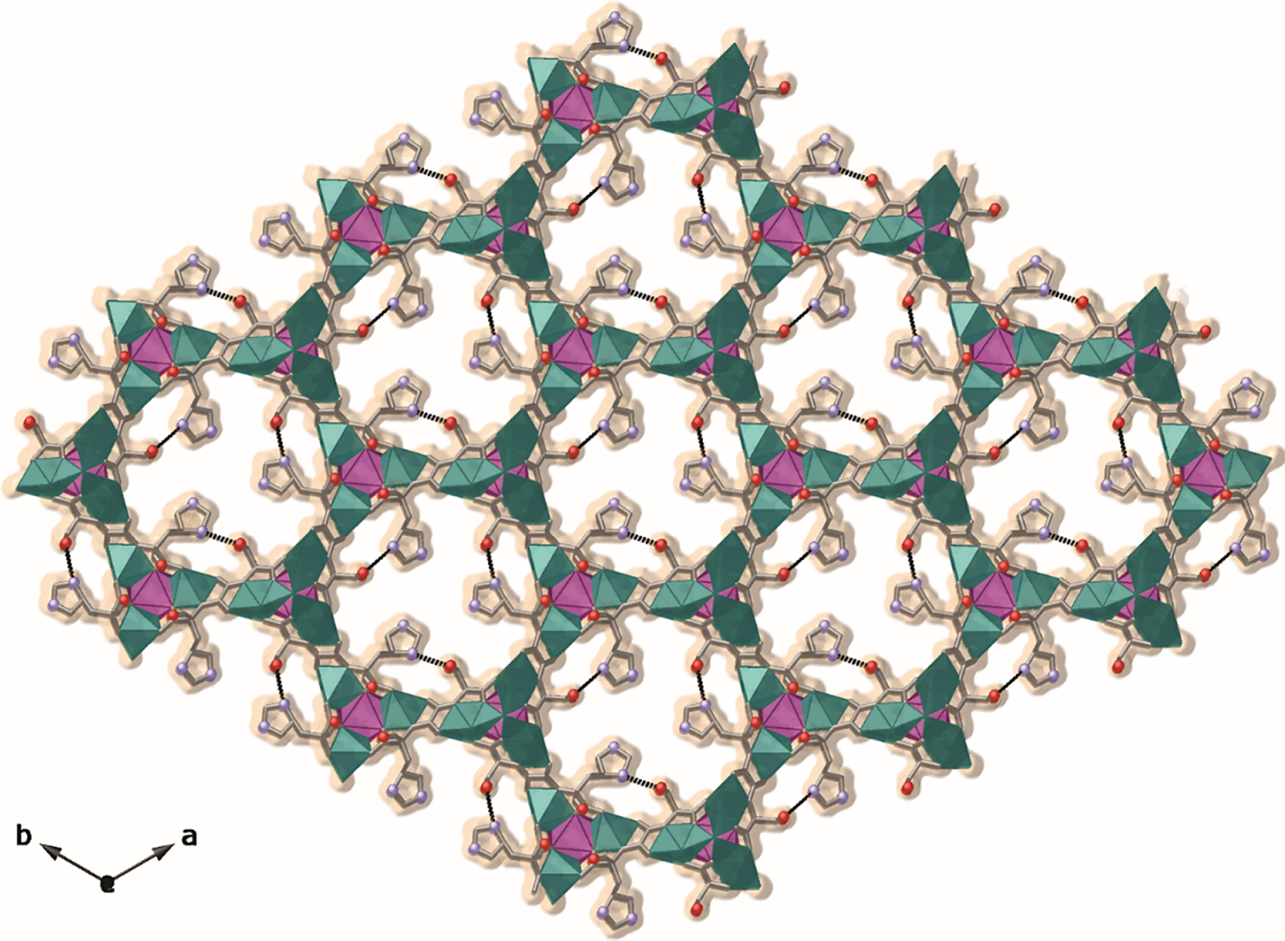
Perspective view of the open-framework of MTV-bioMOF **2** along the *c* axis (the crystallization water molecules are omitted for clarity). Copper(II) and calcium(II) ions from the network are represented as cyan and purple polyhedra whereas organic ligands are depicted as gray sticks with the exception of the oxygen and nitrogen atoms from *L*-serine (−CH_2_OH) and *L*-histidine (−CH_2_C_3_H_3_N_2_) residues, which are represented as red and light blue spheres sticks, respectively. The orange surface represents the van der Waals radii of all metal atoms in order to emphasize the accessible empty space.

**Figure 3:**
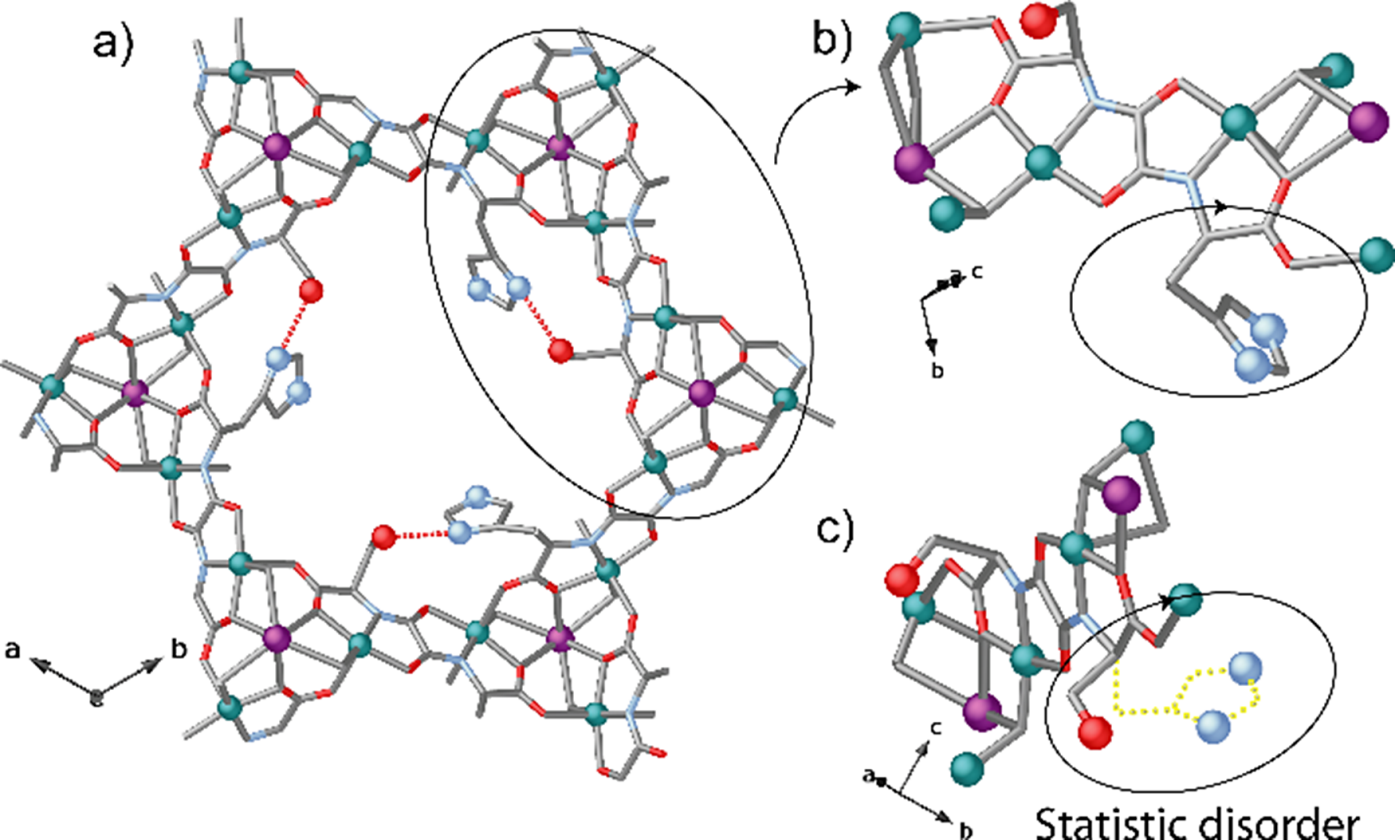
(a) View of a single channel for the porous structure of the MTV-bioMOF **2** along the *c* axis (the crystallization water molecules are omitted for clarity). (b, c) Fragment of the structure of **2** highlighting the dicopper(II) building block and their statistical disorder exhibited by the flexible amino acid residues, −CH_2_OH and −CH_2_C_3_H_3_N_2_, of the serine and histidine, respectively. Copper(II) and calcium(II) ions from the network are represented as cyan and purple spheres, respectively. Oxygen and nitrogen atoms from the residues are shown as red and light blue spheres, respectively. The organic ligands are represented as sticks with the following color scheme: oxygen, red; nitrogen, light blue; carbon, gray.

Well-shaped crystals of MTV-bioMOF **2** were obtained with a slow diffusion technique by mixing stoichiometric amounts (2:1) of Cu(II) precursors with both metalloligands (see the Experimental Section for further details) and thus, the crystal structure of **2** could be unveiled by single-crystal X-ray diffraction (SCXRD) performed using synchrotron radiation at the I19 beamline of the Diamond Light Source (see Supporting Information). **2** crystallizes in the *P*(*−*6) space group and its structure consists of uninodal **acs** six-connected 3D calcium(II)–copper(II) networks featuring functional hexagonal channels, where both types of flexible amino acid residues, −CH_2_OH and −CH_2_C_3_H_3_N_2_, of the serine and histidine, respectively coexist within the channels (Figures 2 and S1–S4). The six-connected networks are assembled by trans-oxamidato-bridged dicopper(II) units of {Cu^II^_2_[(S,S)-serimox]} and {Cu^II^_2_[(S,S)-hismox]}, which are statistically disordered in the crystal structure (Scheme S1 and Figure 3). In **2**, copper(II) dimers act as metallolinkers between the Ca^II^ ions through the carboxylate groups (Figures 3 and S2). Aqua/hydroxo groups (in a 1:2 statistical distribution) further interconnect neighboring Cu^2+^ and Cu^2+^/Ca^2+^ ions whose result is linked in a μ_3_ fashion (Figures S1 and S2).

The high quality of the data set, acquired under synchrotron radiation, allowed the achievement of a final model, appropriate to describe the mixed-ligand structure. Indeed, crystallography answered the expected situation within crystals of MTV-bioMOF **2**, where a statistically disordered distribution of serine and histidine moieties with 67 and 33% occupancy within the network has been detected, respectively (see Crystallographic details in the Supporting Information). The statistically disordered amino acid residues remain confined in the hexagonal pores. The main intrinsic feature of **2** is the intraligand hydrogen bond interaction involving the oxygen atom of the serine and the nitrogen atom of the histidine moieties, respectively (Figures 2 and 3a) [N_his_···O_ser_ 2.783(7) Å]. The noncovalent confined ser-his bioassembly mimics the active site of the catalase enzymes. Indeed, enlightenment of amino acid sequences, resulting from an inspection of several crystal structures of biological materials, shows the existence of a hydrogen bond between the distal His and an Asn or other amino acids. Thus, the catalytic activity of the distal site serine-histidine couple in **2** can be likely related to the local H-bonding interaction, as it is well-established that the catalase activity (of catalase-peroxidases) is modulated by changes in the p*K*_a_ of the distal histidine.^60^

Finally, an extended network of hydrogen bonds involving lattice water molecules further stabilizes the flexible amino acid derivative chains (Figure S4).

The higher percentage of serimox respect to hismox leads to dominant {Ca^II^Cu^II^_6_[(*S*,*S*)-serimox] superimposed for 33% of unit cells with {Cu^II^_2_[(S,S)-hismox]}, and thus, resulting in a snapshot of mixed [Ca^II^Cu^II^_6_[(*S*,*S*)-serimox/hismox] fragments (Figure 3c). The mixed percentage has been further confirmed by the composition analysis (vide infra C, H, S, and N; Supporting Information). This disorder gives an averaged view of **2** with a crystal structure that is, of course, the spatial average, of all molecules/fragments, together with all their possible orientations averaged, in the crystal via only one unit cell.

The multivariate structure is still highly hydrophilic and porous (Figure S3), comparable with that of the single-ligand parent compound serimox as confirmed by thermogravimetric analysis (TGA) (Figure S5) and the N_2_ adsorption isotherms at 77 K of **2** (Figure S6).

The chemical nature of the novel MTV-bioMOF **2**, determined by SCXRD, was further confirmed by C, H, S, N, and scanning electron microscopy with energy dispersive X-ray spectroscopy (SEM-EDX) analyses (see Tables S2–S5 and chemical formulas in Experimental Section), which corresponds exactly to a chemical composition with 33 and 67% of *L*-histidine and *L*-serine, respectively, independently of the precursors ratio used. In addition, these analyses were carried out for both individual monocrystals (Table S2) and the bulk polycrystalline samples (Tables S3–S5), showing identical results. Thus, confirming that MTV-bioMOF **2** has the same ligand ratio composition (1:2) and is identical to that present in the reaction mixture. This demonstrates that there are significant ligand preferences, which most likely could be related to steric and stabilization effects. SEM-EDX elemental mappings for Cu, Ca, N, and O elements (Figure S7) confirm homogeneous spatial distributions of all elements through the bioMOFs **1** (Figure S7a) and **2** (Figure S7b) samples.

TGA (Figure S5) of **2** allowed us to estimate the number of crystallization water molecules (see chemical formula in Experimental Section). The experimental powder X-ray diffraction (PXRD) patterns of polycrystalline samples of **2** confirm both that bulk samples are identical to the crystal selected for SCXRD and also the homogeneity of the polycrystalline samples (Figure S8). Finally, the N_2_ adsorption isotherms at 77 K of **2** (Figure S6) confirmed its permanent porosity, which is important in aiming to carry out successful catalytic experiments. In this sense, the calculated Brunauer–Emmett–Teller (BET) surface area^61^ for **2** is 438.7 m^2^/g.

**Figure 4:**
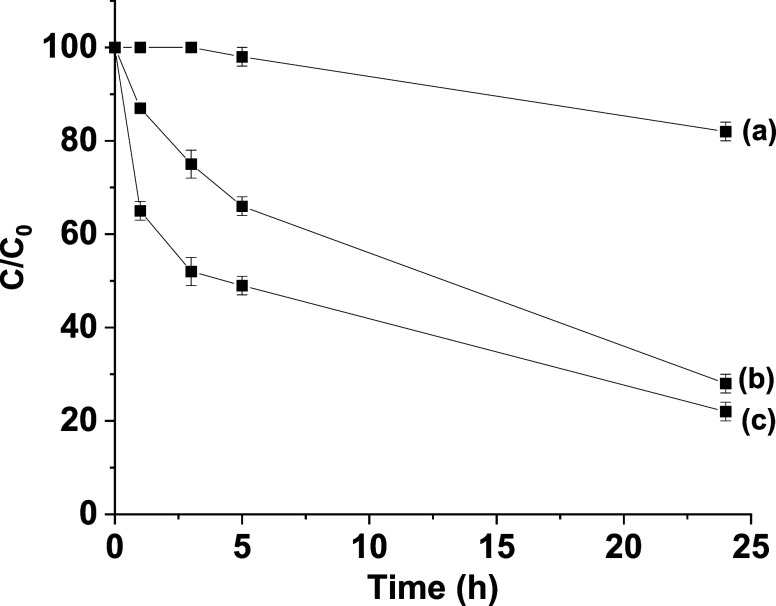
Catalase-like performance of **1** and **2** solids in the decomposition of H_2_O_2_ at 37 °C. These data correspond to the three independent experiments. Legend: (a) absence of catalyst; (b) catalyst **1**; (c) catalyst **2**.

Aiming at evaluating the catalase-like enzymatic activity, we measured the decomposition of hydrogen peroxide aqueous solutions in the presence of bioMOF **1** and MTV-bioMOF **2**, separately (Figure 4).

A control experiment in the absence of a catalyst resulted in 18% degradation of H_2_O_2_, which is relatively lower compared to the activity of **1** and **2**. Among the solids **1** and **2**, the catalase-like activity was relatively superior for **2** compared to **1**. As commented earlier, the superior performance of **2** is most likely due to the presence of 33% of histidine residues (−CH_2_C_3_H_3_N_2_) decorating the pores, thus mimicking the role of the catalase enzyme.^60^ These results suggest the occurrence of a histidine-mediated mechanism, as depicted in Scheme S2a. In contrast, the inferior performance of **1** can be attributed to the lack of histidine residues, and the catalytic activity arises only from the copper(II) centers anchored within the framework. These catalytic data indicate and support the catalase-mimicking nature of bioMOFs through their appropriate design and tunability. Furthermore, the stability of bioMOF **1** and MTV-bioMOF **2** was assessed in three consecutive cycles and the observed data are shown in Figures S9 and S10. Interestingly, the activity of both solids was retained for three cycles with no significant decrease in their activities. The performance of MTV-bioMOF **2** after 24 h in the first, second, and third runs was 82, 84, and 76% respectively, while for bioMOF **1** after 24 h was 75, 76, and 72% for the first, second and third runs, respectively. Finally, PXRD patterns of bioMOF **1** and MTV-bioMOF **2**, after catalytic experiments (Figure S11), confirm that the samples remain crystalline.

## Conclusions

In summary, we have first evaluated the catalase activity of a previously reported amino-acid based copper(II)–calcium(II) bioMOF, with formula {Ca^II^Cu^II^_6_[(*S*,*S*)-serimox]_3_(OH)_2_(H_2_O)}·39H_2_O (**1**), possessing functional channels densely decorated with −CH_2_OH groups and accessible copper(II) centers, whose coordination environment is reminiscent of those observed in other copper(II) compounds exhibiting certain activity in the degradation of hydrogen peroxide to water and oxygen.^62^ As a result, **1** exhibits moderate enzymatic activity. With these results in hand, we then devoted our efforts to the design and preparation of a novel MTV-bioMOF, combining both *L*-serine- and *L*-histidine-derived ligands within the open framework. The resulting MTV-bioMOF, with formula Ca^II^Cu^II^_6_[(*S*,*S*)-serimox]_2_[(*S*,*S*)-hismox]_1_(OH)_2_(H_2_O)}·27H_2_O (**2**), exhibits improved catalase-like enzymatic catalysis compared to bioMOF **1**, somehow confirming the key role play by histidine residues in the hydrogen peroxide decomposition, and suggesting an histidine-mediated mechanism instead of the direct one (Scheme S2).^60^ Overall, this work constitutes an step forward on the rational design MTV-bioMOFs acting as enzyme mimics, where the heterogeneity provided by the different functional groups provides the tools to emulate active centers in enzymes.

## Experimental Section

### {Ca^II^Cu^II^_6_[(*S*,*S*)-serimox]_2_[(*S*,*S*)-hismox]_1_(OH)_2_(H_2_O)}·27H_2_O (**2**)

Well-shaped hexagonal prisms of **2** suitable for SCXRD were obtained by slow diffusion in H-shaped tubes of water/methanol (9:1) solutions containing stoichiometric amounts (2:1) of (Me_4_N)_2_{Cu_2_[(*S*,*S*)-serimox](OH)_2_}·5H_2_O (0.158 g, 0.24 mmol) and (Me_4_N)_2_{Cu_2_[(*S*,*S*)-hismox](OH)_2_}·4H_2_O (0.10 g, 0.12 mmol) in one arm and CaCl_2_·2H_2_O (0.018 g, 0.12 mmol) in the other. They were isolated by filtration on paper and air-dried. Anal. Calcd for **2**: C_30_Cu_6_CaH_86_N_10_O_52_ (1840.4): C, 19.58; H, 4.71; N, 7.61%. Found: C, 19.63; H, 4.67; N, 7.64%; IR (KBr): ν = 1608 and 1603 cm^–1^ (C=O). C, H, N, analyses and TGA analyses gave a final formula of {Ca^II^Cu^II^_6_[(*S,S*)-serimox]_2_(*S,S*)-hismox]_1_(OH)_2_(H_2_O)}·27H_2_O.

A gram-scale procedure was also carried out successfully by mixing greater amounts of (Me_4_N)_2_{Cu_2_[(S,S)-serimox](OH)_2_}·5H_2_O (3.166 g, 4.80 mmol) and (Me_4_N)_2_{Cu_2_[(S,S)-hismox](OH)_2_}·4H_2_O (1.747 g, 2.40 mmol) in water (40 mL). Another aqueous solution of CaCl_2_·2H_2_O (0.176 g, 1.20 mmol) was added dropwise to the resulting deep green solution and the final mix was allowed to react, under stirring, for 6 h. Afterward, the material was isolated by filtration and characterized by C, H, N analyses and TGA analyses to give a final formula of Ca^II^Cu^II^_6_[(*S,S*)-serimox]_2_(*S,S*)-hismox]_1_(OH)_2_(H_2_O)}·27H_2_O (**2**). Yield: 2.00 g, 87%; Anal. calcd for **2**: C_30_Cu_6_CaH_86_N_10_O_52_ (1840.4): C, 19.58; H, 4.71; N, 7.61%. Found: C, 19.48; H, 4.61; N, 7.69%; IR (KBr): ν = 1609 and 1603 cm^–1^ (C=O).

### Gas Adsorption

The N_2_ adsorption–desorption isotherms at 77 K, were carried out on a polycrystalline sample of **2** with a BELSORP-mini-X instrument. The sample was first activated with methanol and then evacuated at 348 K during 19 h under 10^–6^ Torr prior to their analysis.

### Microscopy Measurements

SEM elemental analysis was carried out for **2**, for both powdered samples and single crystals, using a HITACHI S-4800 electron microscope coupled with an Energy Dispersive X-ray (EDX) detector. Data was analyzed with QUANTAX 400.

### X-ray Powder Diffraction Measurements

A polycrystalline sample of **2** was introduced into a 0.5 mm borosilicate capillary prior to being mounted and aligned on an Empyrean PANalytical powder diffractometer, using Cu Kα radiation (λ = 1.54056 ÅFive repeated measurements were collected at room temperature (2θ = 2–60°) and merged in a single diffractogram. PXRD patterns of solid polycrystalline samples **1** and **2** were also obtained after the catalytic experiments with the same equipment.

### X-ray Crystallographic Data Collection and Structure Refinement

A crystal of **2** with ca. 0.12 × 0.10 × 0.10 mm as dimensions was selected and mounted on a MITIGEN holder in Paratone oil and very quickly placed on a liquid nitrogen stream cooled at 90 K to avoid the possible degradation upon dehydration. Diffraction data for **2** were collected using a synchrotron at the I19 beamline of the DIAMOND at λ = 0.6889 Å. Further crystallographic details can be found in the Supporting Information.

### General Catalytic Reaction Procedure

Hydrogen peroxide decomposition was conducted in buffered aqueous solution at 37 °C. One phosphate-buffered saline (PBS) tablet (Sigma-Aldrich, ref P4417-50TAB) was dissolved in Milli-Q water (200 mL). This solution is called PBS water. On the other hand, 1 mL of 30% H_2_O_2_ and 9 mL of Milli-Q water were mixed as a stock solution. In a typical experiment, a glass container was charged with 10 mg of **1** or **2** followed by 50 mL of PBS water and incubated for 2 h at 37 °C. After this, 0.15 mL of H_2_O_2_ stock solution was added to the reaction mixture containing **1** or **2** solids. The progress of the H_2_O_2_ decomposition was monitored by sampling aliquots of 5 mL from the reaction mixture, diluted by 10-fold and an indicator of K_2_(TiO)(C_2_O_4_)_2_ in H_2_SO_4_/HNO_3_ was added. The concentration of H_2_O_2_ was monitored by a colorimetric method at 420 nm using a Jasco UV–visible spectrophotometer with a fixed wavelength mode. Degradation at different time intervals was performed in the range of 200–600 nm. Reusability experiments were performed identically as described above. After the reaction, the solid was recovered by centrifugation at 10,000 rpm at 10 °C, washed with Milli-Q water, and collected. This recovered solid was used for consecutive cycles. Further details can be found in the Supporting Information.

## Supporting Information Available

Additional experimental preparation details and analytical characterization with Tables S1–S5, Schemes S1–S2, and Figures S1–S11 (PDF)
