# Supplementary material for: Design of Multivariate Biological Metal–Organic Frameworks: Toward Mimicking Active Sites of Enzymes
Source: Inorg Chem. 2024 Jul 9;63(29):13681–8. doi: 10.1021/acs.inorgchem.4c01988 (PMC11271005; doi:10.1021/acs.inorgchem.4c01988)
Supplement: Supplementary file 1 — ic4c01988_si_001.pdf [file ic4c01988_si_001.pdf]

Supporting Information (ESI) for the manuscript:

Design of Multivariate Biological Metal-Organic Frameworks: Towards Mimicking active sites of Enzymes

Javier Navarro-Alapont,[a] Cristina Negro,[a] Sergio Navalón,[b] Amarajothi Dhakshinamoorthy,[b] Donatella Armentano,[c] Jesús Ferrando-Soria,[a] and Emilio Pardo*[a]

AUTHOR ADDRESSES

[a]Instituto de Ciencia Molecular (ICMol), Universidad de Valencia, 46980 Paterna, Valencia, Spain

[b]Departamento de Química, Universitat Politècnica de València, Camino de Vera s/n, Valencia 46022, Spain.

[c]Dipartimento di Chimica e Tecnologie Chimiche (CTC), Università della Calabria, Rende 87036, Cosenza, Italy

Email: donatella.armentano@unical.it; emilio.pardo@uv.es

Experimental Section

Materials. All chemicals were of reagent grade quality. They were purchased from commercial sources and used as received. Organic ligands [H_2Me_2-(*S*,*S*)-hismox and H_2Me_2-(*S*,*S*)-serimox], dinuclear precursor complex [$(Me_4N)_2\{Cu_2$[(*S*,*S*)-hismox]$(OH)_2\}$ · $4H_2O$ and $(Me_4N)_2\{Cu_2$[(S,S)-serimox]$(OH)_2\}$ · $5H_2O$] and the isoreticular MOF $\{Ca^{II}Cu^{II}_6$[(*S*,*S*)-serimox]$_3(OH)_2(H_2O)\}$ · $39H_2O$ (**1**) were prepared as previously reported.[1,2]

$\{Ca^{II}Cu^{II}_6$[(*S*,*S*)-serimox]$_2$[(*S*,*S*)-hismox]$_1(OH)_2(H_2O)\}$ · $27H_2O$ (2): Well-shaped hexagonal prisms of **2** suitable for SCXRD were obtained by slow diffusion in H-shaped tubes of water/methanol (9:1) solutions containing stoichiometric amounts (2:1) of $(Me_4N)_2\{Cu_2$[(S,S)-serimox]$(OH)_2\}$ · $5H_2O$ (0.158 g, 0.24 mmol) and $(Me_4N)_2\{Cu_2$[(S,S)-hismox]$(OH)_2\}$ · $4H_2O$ (0.10 g, 0.12 mmol) in one arm and $CaCl_2$ · $2H_2O$ (0.018 g, 0.12 mmol) in the other. They were isolated by filtration on paper and air-dried. Anal. calcd for **2**: $C_{30}Cu_6CaH_{86}N_{10}O_{52}$ (1840.4): C, 19.58; H, 4.71; N, 7.61%. Found: C, 19.63; H, 4.67; N, 7.64%; IR (KBr): ν = 1608 and 1603 cm^{-1} (C=O). C, H, N, analyses and TGA analyses gave a final formula of $\{Ca^{II}Cu^{II}_6$[(*S*,*S*)-serimox]$_2$(*S*,*S*)-hismox]$_1(OH)_2(H_2O)\}$ · $27H_2O$.

A gram-scale procedure was also carried out successfully by mixing greater amounts of $(Me_4N)_2\{Cu_2$[(S,S)-serimox]$(OH)_2\}$ · $5H_2O$ (3.166 g, 4.80 mmol) and $(Me_4N)_2\{Cu_2$[(S,S)-hismox]$(OH)_2\}$ · $4H_2O$ (1.747 g, 2.40 mmol) in water (40 mL). Another aqueous solution of $CaCl_2$ · $2H_2O$ (0.176 g, 1.20 mmol) was added dropwise to the resulting deep green solution and the final mix was allowed to react, under stirring, for 6 hours. Afterwards, the material was isolated by filtration and characterized by C, H, N analyses and TGA analyses to give a final formula of $\{Ca^{II}Cu^{II}_6$[(*S*,*S*)-serimox]$_2$(*S*,*S*)-hismox]$(OH)_2(H_2O)\}$ · $27H_2O$ (**4**). Yield: 2.00 g, 87%; Anal. calcd for **2**:

$C_{30}Cu_6CaH_{86}N_{10}O_{52}$ (1840.4): C, 19.58; H, 4.71; N, 7.61%. Found: C, 19.48; H, 4.61; N, 7.69%; IR (KBr): ν = 1609 and 1603 cm^{-1} (C=O).

Physical Techniques: Elemental (C, H, N) analyses were performed at the Microanalytical Service of the Universitat de València. FT–IR spectra were recorded on a Perkin-Elmer 882 spectrophotometer as KBr pellets. The thermogravimetric analyses were performed on crystalline samples under a dry N_2 atmosphere with a Mettler Toledo TGA/STDA 851e thermobalance operating at a heating rate of 10 ºC min^{-1}.

The N_2 adsorption-desorption isotherms at 77 K were carried out on crystalline samples of **1** and **2** with a BELSORP MINI X instrument. Samples were activated at 70 °C under reduced pressure (10^{-6} Torr) for 16 h prior to carry out the sorption measurements.

X-ray crystallographic data collection and structure refinement: A crystal of **2** with 0.12 x 0.10 x 0.10 mm was selected and mounted on a MITIGEN holder in Paratone oil and very quickly placed on a liquid nitrogen stream cooled at 30 K to avoid the possible degradation upon dehydration. Diffraction data for **2** was collected using synchrotron radiation at I19 beamlines in DIAMOND (λ = 0.6889 Å). The data were processed through xia2 software.[3] The structure was solved with the SHELXS structure solution program, using the Patterson method. The model was refined with version SHELXL-2019/1 against F^2 on all data by full–matrix least squares.[4]

All non–hydrogen atoms of the MOF network, except some highly dynamically disordered atoms belonging to the amino acidic residues confined in pores (dynamical fragments of the structure) and lattice water molecules found, were refined anisotropically. The use of some C–C bond lengths restrains and rigid groups, during the refinements in **2**, has been reasonable imposed and related to extraordinary

flexibility of terminal histidine and serine moieties of the ligand residing in large pores. In the refinement of **2** crystal structure, some further restrains, to make the refinement more efficient, have been applied. For instance, ADP components have been restrained to be similar to other related atoms, using EADP for group of atoms of the histidine rigid group expected to have essentially similar ADPs.

In **2**, all the hydrogen atoms of the networks were set in calculated position and refined isotropically using the riding model except for serine and histidine fragments and for found solvent lattice molecules where they were neither found nor calculated. These molecules are expected to be severely disordered as a direct consequence of their high thermal motion and exhibit statistic disorder. As stated in main text, the oxamidato-bridged dicopper(II) units of {Cu^{II}_2[(S,S)-serimox]} and {Cu^{II}_2[(S,S)-hismox]} unveiled with a 67:33 in % ratio in **2** exhibit a statistically disorder in the crystal structure (Figure 3c), where the higher percentage of serimox versus hismox leads to a completely superimposed snapshot of mixed {Cu^{II}_2[(S,S)- serimox/hismox]} dimers. In fact, such a disorder gives a mixed view of **2,** understandable considering that a crystal structure is the *spatial average*, of all molecules/fragments, together with all their possible orientations averaged, in the crystal *via* only one unit cell.

The solvent water molecules (and also one methanol molecule found in the structure) were severely disordered but, even if not all the ones detected by TGA analysis, have been modelled. Because of that, the mismatches between calculated and reported formulae are detected as Alerts of Level A in checkcifs because in the final formulae all the solvent molecules, confirmed by TGA, have been reported.

The comments for the main alerts A and B are described in the CIF using the validation reply form (vrf).

Without found solvent molecules, the effective free volume of **2** is calculated by PLATON analysis to be 42.8% of the crystal volume (1472 Å^3 of the 3442.0 Å^3 of the unit cell volume). In accordance with BET and SCXRD analysis, the channels of **2** are almost entirely filled by serine and histidine amino acid fragments strongly enlaced by hydrogen bond interactions together with solvent molecules (water and methanol).

A summary of the crystallographic data and structure refinement for **2** is given in Table S1.

The final geometrical calculations on free voids and the graphical manipulations were carried out with PLATON[5] implemented in WinGX,[6] and CRYSTAL MAKER programs,[7] respectively. Data have been deposited at Cambridge Crystallographic Database with CCDC code 2320455.

X-ray Powder Diffraction Measurements: A fresh polycrystalline sample of **2** was introduced into 0.5 mm borosilicate capillaries prior to being mounted and aligned on a Empyrean PANalytical powder diffractometer, using Cu Kα radiation (λ = 1.54056 Å). Five repeated measurements were collected at room temperature (2θ = 2–60°) and merged in a single diffractogram. PXRD patterns of solid polycrystalline samples of **1** and **2** were also obtained after the catalytic experiments with the same Empyrean PANalytical powder diffractometer, using Cu Kα radiation (λ = 1.54056 Å) at room temperature (2θ = 2–60°).

Catalysis details: Hydrogen peroxide decomposition was conducted in buffered aqueous solution at 37 ºC. One phosphate buffered saline (PBS) tablet (Sigma Aldrich, ref. P4417-50TAB) was dissolved in MilliQ water (200 mL). This solution is called as PBS-water. On the other hand, 1 mL 30% H_2O_2 and 9 mL of MilliQ water were mixed as a stock solution. In a typical experiment, a glass container was charged with 10 mg of **1** or **2** followed by 50 mL of PBS-water and incubated for 2 h at 37 ºC. After this, 0.15

mL of H_2O_2 stock solution was added to the reaction mixture containing **1** or **2** solids. The progress of the H_2O_2 decomposition was monitored by sampling aliquots of 5 mL from the reaction mixture, diluted by ten-fold and an indicator of $K_2(TiO)(C_2O_4)_2$ in H_2SO_4/HNO_3 was added. The concentration of H_2O_2 was monitored by a colorimetric method at 420 nm using Jasco UV-Visible spectrophotometer with a fixed wavelength mode.[8]

Table S1. **Summary of Crystallographic Data for 2**

Compound	**2**
Formula	C_{30} $H_{86}CaCu_6N_{10}O_{52}$
M (g mol^{-1})	1840.40
λ (Å)	0.68890
Crystal system	hexagonal
Space group	P(-6)
a (Å)	17.79080(10)
c (Å)	12.55760(10)
V (Å^3)	3442.14(5)
Z	2
ρ_{calc} (g cm^{-3})	1.776
μ (mm^{-1})	1.778
T (K)	100
θ range for data collection (°)	1.572 to 27.000
Completeness to θ = 25.0	100%
Measured reflections	51600
Unique reflections (Rint)	5778 (0.0447)
Observed reflections [$I > 2\sigma(I)$]	5054
Goof	1.167
Absolute structure parameter (Flack)	0.48(5)
R^a [$I > 2\sigma(I)$] (all data)	0.0660 (0.0710)
wR^b [$I > 2\sigma(I)$] (all data)	0.1994 (0.2044)

^{a} $R = \sum(|F_o| - |F_c|)/\sum|F_o|$. ^{b} $wR = [\sum w(|F_o| - |F_c|)^2/\sum w|F_o|^2]^{1/2}$.

Table S2. Selected data from the Elemental (C, H, N)[b] and SEM/EDX[b] analyses on single crystals of **2**.

**Element**	**Percentage (%)**
C	19.51[a]
H	4.63[a]
N	7.69[a]
Cu	20.9[b]
Ca	2.1[b]

Table S3. Selected data from the Elemental (C, H, N)[a] and SEM/EDX[b] analyses on a powdered polycrystalline sample of **2**, prepared by following the synthetic procedure reported in the Experimental Section (serine/histidine ratio = 2:1).

**Element**	**Percentage (%)**
C	19.59[a]
H	4.39[a]
N	7.69[a]
Cu	20.5[b]
Ca	2.3[b]

Table S4. Selected data from the Elemental (C, H, N)[a] and SEM/EDX[b] analyses on a powdered polycrystalline sample of **2,** prepared by following a synthetic procedure similar to that reported in the Experimental Section but using a 1:1 serine/histidine ratio.

**Element**	**Percentage (%)**
C	19.63[a]
H	4.61[a]
N	7.62[a]
Cu	20.8[b]
Ca	2.3[b]

Table S5. Selected data from the Elemental (C, H, N)[a] and SEM/EDX[b] analyses on a powdered polycrystalline sample of **2,** prepared by following a synthetic procedure similar to that reported in the Experimental Section but using a 1:2 serine/histidine ratio.

**Element**	**Percentage (%)**
C	19.54[a]
H	4.49[a]
N	7.62[a]
Cu	20.7[b]
Ca	2.1[b]

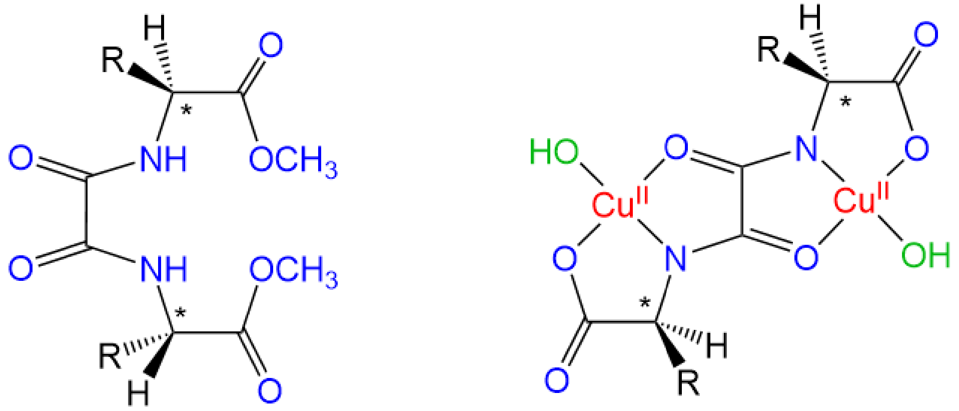

H_2Me_2L $[Cu^{II}_2L(OH_2)]^{2-}$

R = $-CH_2OH$	H_2Me_2-(S,S)-serimox	$\{Cu_2[(S,S)\text{-serimox}](OH)_2\}^{2-}$
R = $-CH_2SC_3H_3N_2$	H_2Me_2-(S,S)-hismox	$\{Cu_2[(S,S)\text{-hismox}](OH)_2\}^{2-}$

Scheme S1. Chemical structures of the chiral bis(amino acid)oxalamide ligands (left), highlighting the potential coordination sites and chiral centers (*) and the corresponding dianionic bis(hydroxo) dicopper(II) complexes, which constitute the secondary building units (SBUs) in the MOF (right).

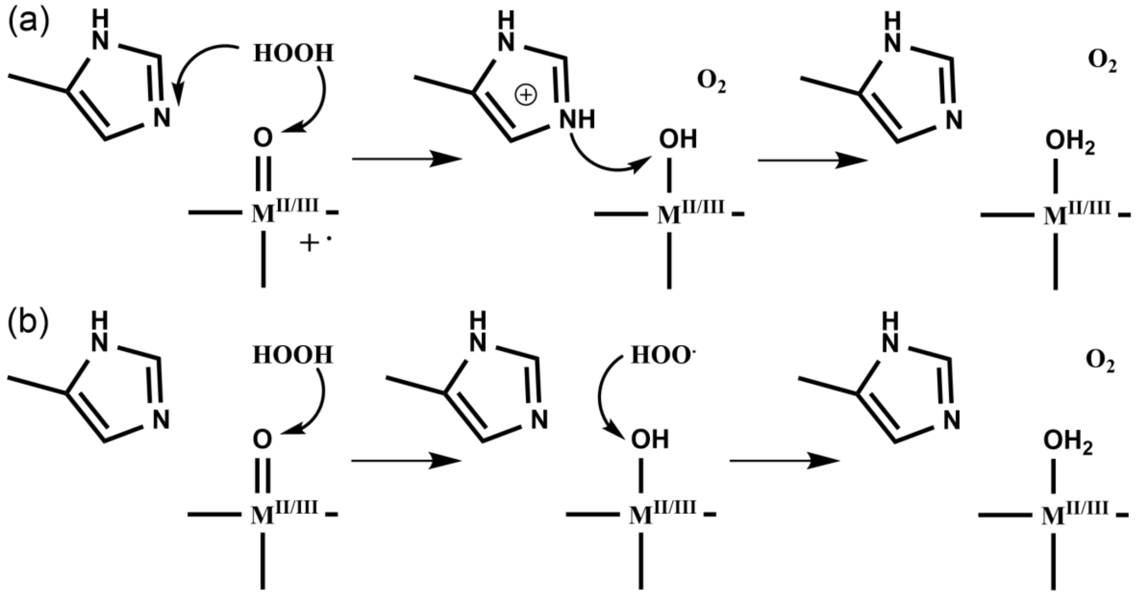

Scheme S2. Two plausible mechanisms for the catalase reaction as suggested in reference 60: a *His-mediated* mechanism (a) and a *direct* mechanism (b) with no participation of histidines.

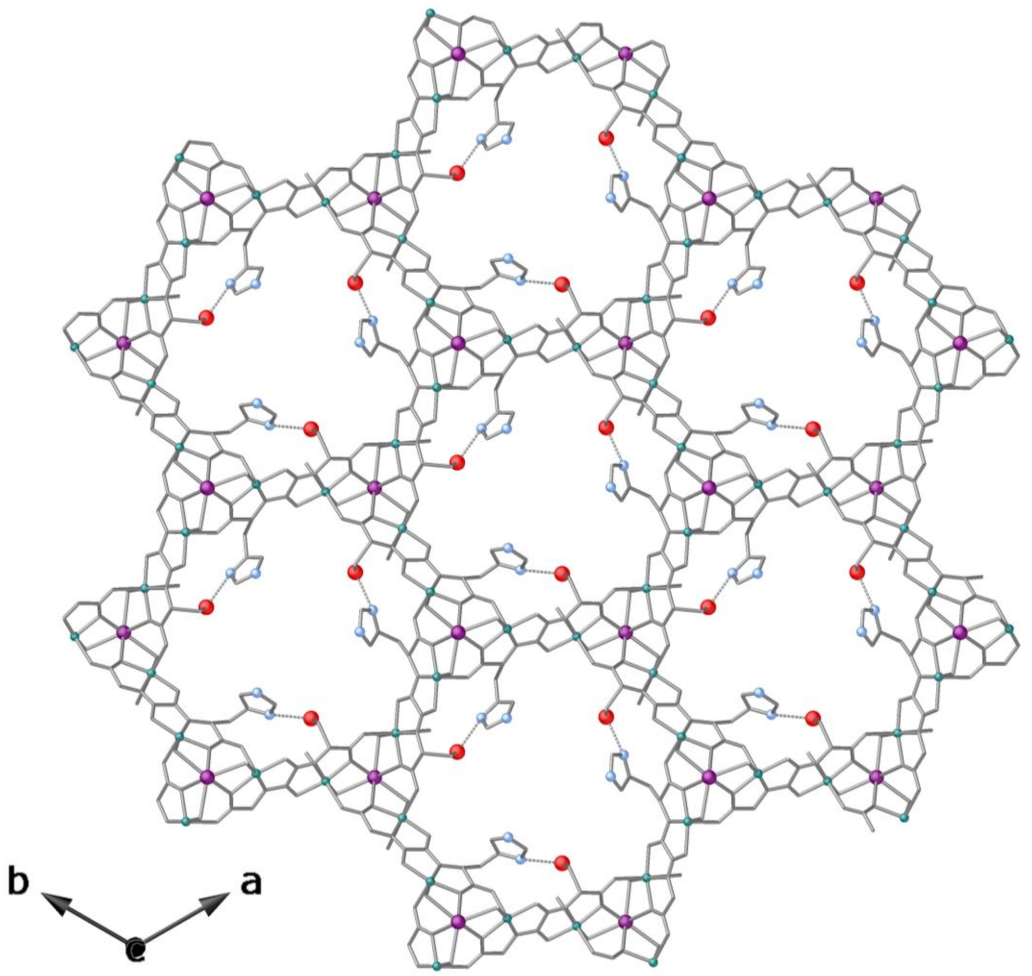

Figure S1. View of the porous structure of $\{Ca^{II}Cu^{II}_6[(S,S)\text{-serimox}]_2[(S,S)\text{-hismox}]_1(OH)_2(H_2O)\} \cdot 27H_2O$ (MTV-bioMOF **2)**, along the *c* axis (the crystallization water molecules are omitted for clarity). Copper and calcium are represented by cyan and purple spheres, respectively. Ligands from the network are depicted as gray stick with the exception of the *L*-serine ($–CH_2OH$) and *L*-histidine ($–CH_2C_3H_3N_2$) residues, which are represented with red and light blue spheres for oxygen and nitrogen atoms, respectively.

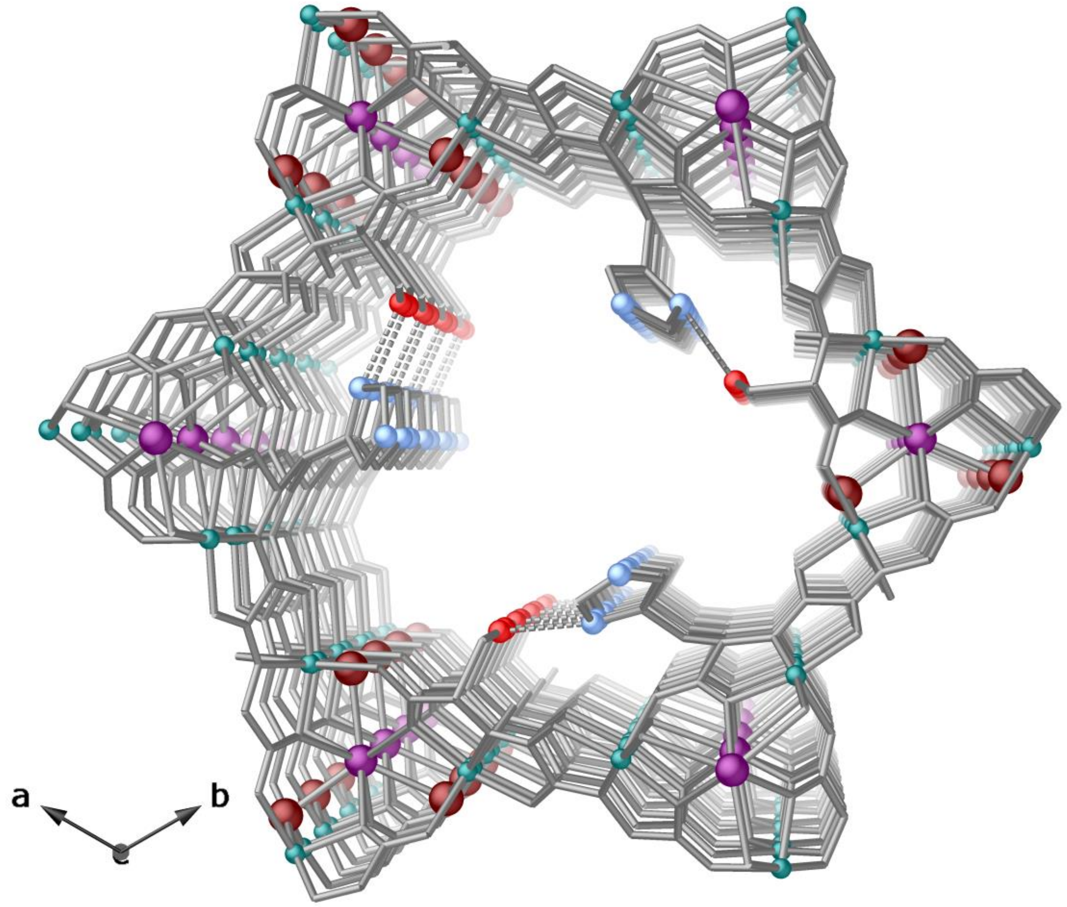

Figure S2. Perspective view of a single channel for the porous structure of the MTV-bioMOF **2** along the *c* axis (the crystallization water molecules are omitted for clarity). Copper and calcium are represented by cyan and purple spheres, respectively. Ligands from the network are depicted as gray stick with the exception of the *L*-serine ($–CH_2OH$) and *L*-histidine ($–CH_2C_3H_3N_2$) residues, which are represented with red and light blue spheres for oxygen and nitrogen atoms, respectively. The oxygen atoms of aqua/hydroxo groups are depicted by deep red spheres.

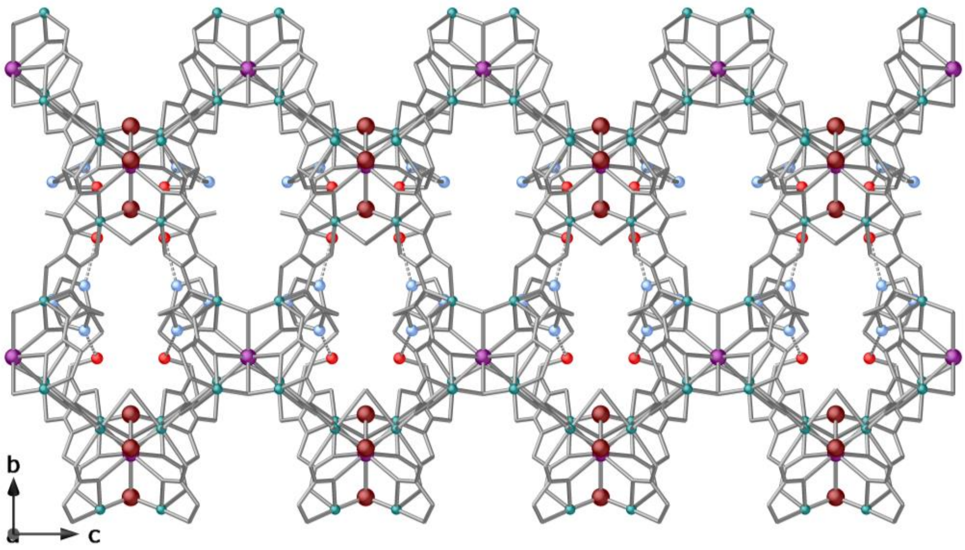

Figure S3. View of a single channel for the porous structure of the MTV-bioMOF **2** along the *a* axis (the crystallization water molecules are omitted for clarity). Copper and calcium are represented by cyan and purple spheres, respectively. Ligands from the network are depicted as gray stick with the exception of the *L*-serine ($–CH_2OH$) and *L*-histidine ($–CH_2C_3H_3N_2$) residues, which are represented with red and light blue spheres for oxygen and nitrogen atoms, respectively. The oxygen atoms of aqua/hydroxo groups are depicted by deep red spheres.

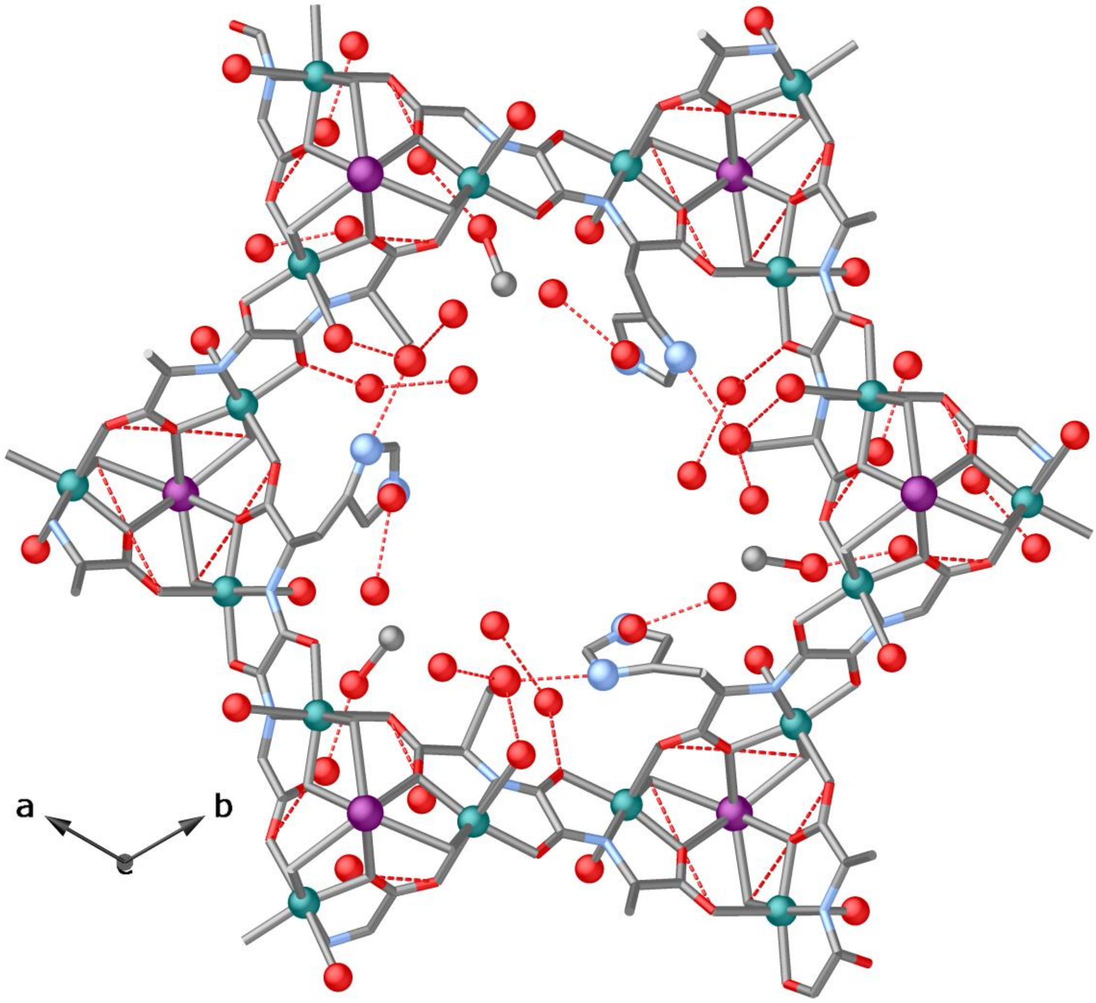

Figure S4. View of a single channel for the porous structure of the MTV-bioMOF **2** along the *a* axis (the crystallization water molecules are omitted for clarity). Copper and calcium are represented by cyan and purple spheres, respectively. Ligands from the network are depicted as gray stick with the exception of the *L*-serine (–CH_2OH) and *L*-histidine (–$CH_2C_3H_3N_2$) residues, which are represented with red and light blue spheres for oxygen and nitrogen atoms, respectively. The oxygen atoms of aqua/hydroxo groups are depicted by deep red spheres.

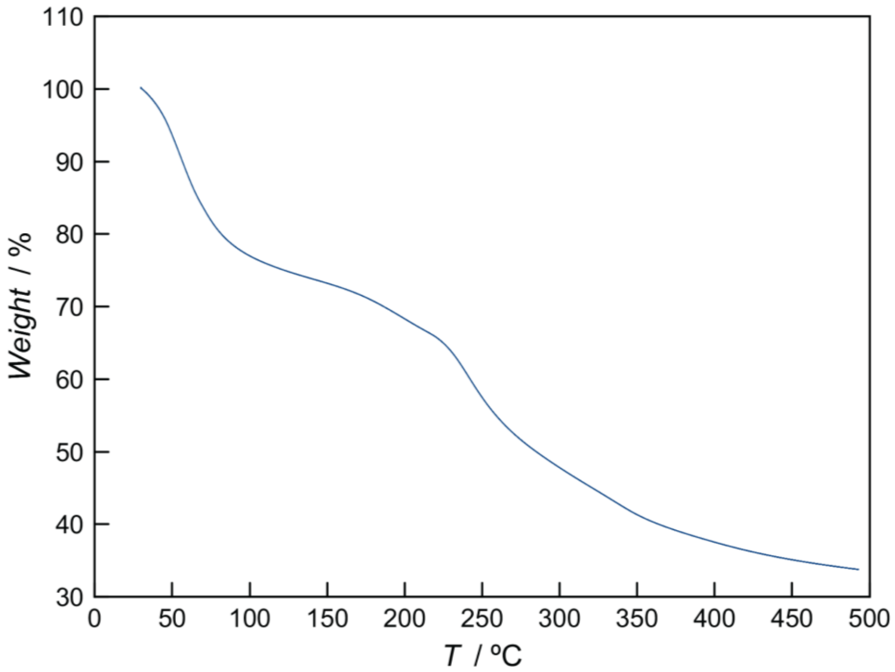

Figure S5. Thermo-Gravimetric Analysis (TGA) of **2** under dry N_2 atmosphere.

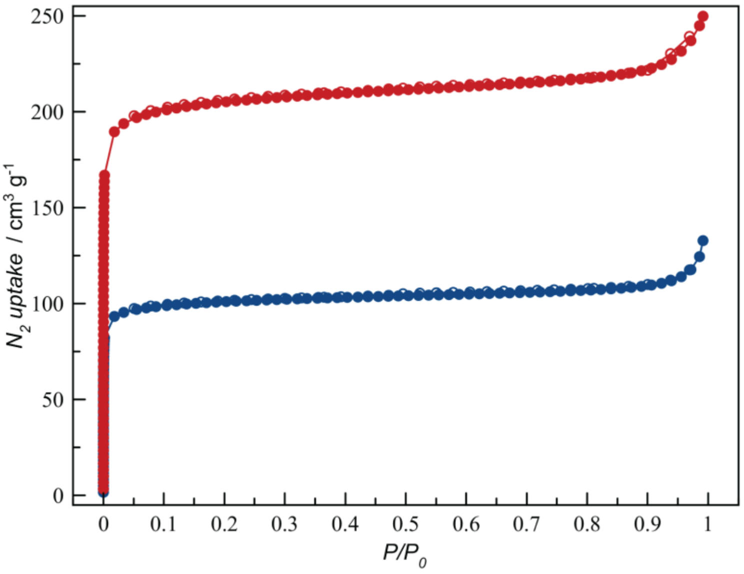

Figure S6. N_2 (77 K) adsorption isotherms for the activated compounds **1** (red) and **2** (blue). Filled and empty symbols indicate the adsorption and desorption isotherms, respectively. The samples were activated at 70 °C under reduced pressure for 16 h prior to carry out the sorption measurements.

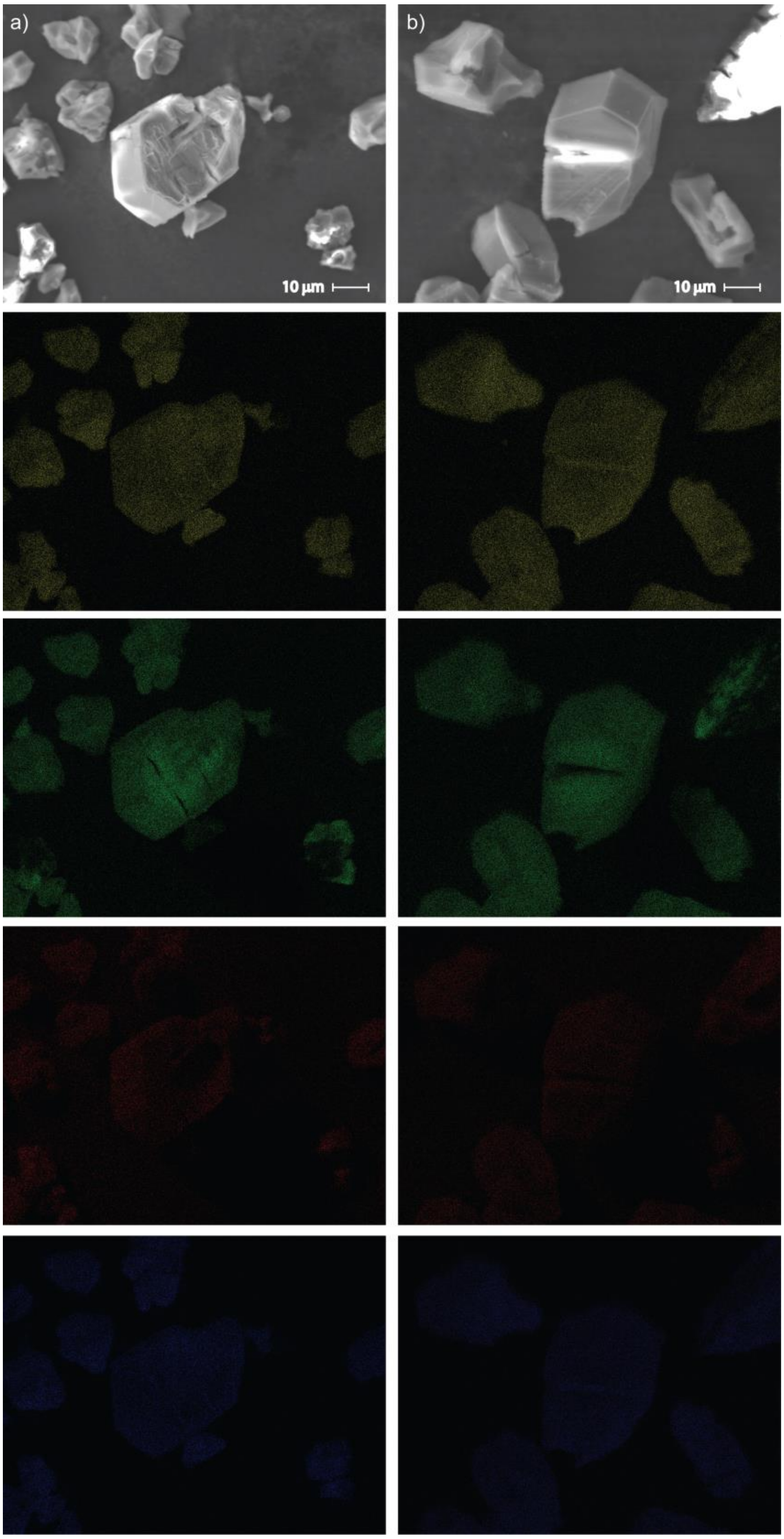

Figure S7. SEM image of a polycrystalline sample of **1** (a) and **2** (b) as well as the corresponding EDX elemental mapping of the bulk for Cu (yellow), Ca (green), N (red) and O (blue) elements.

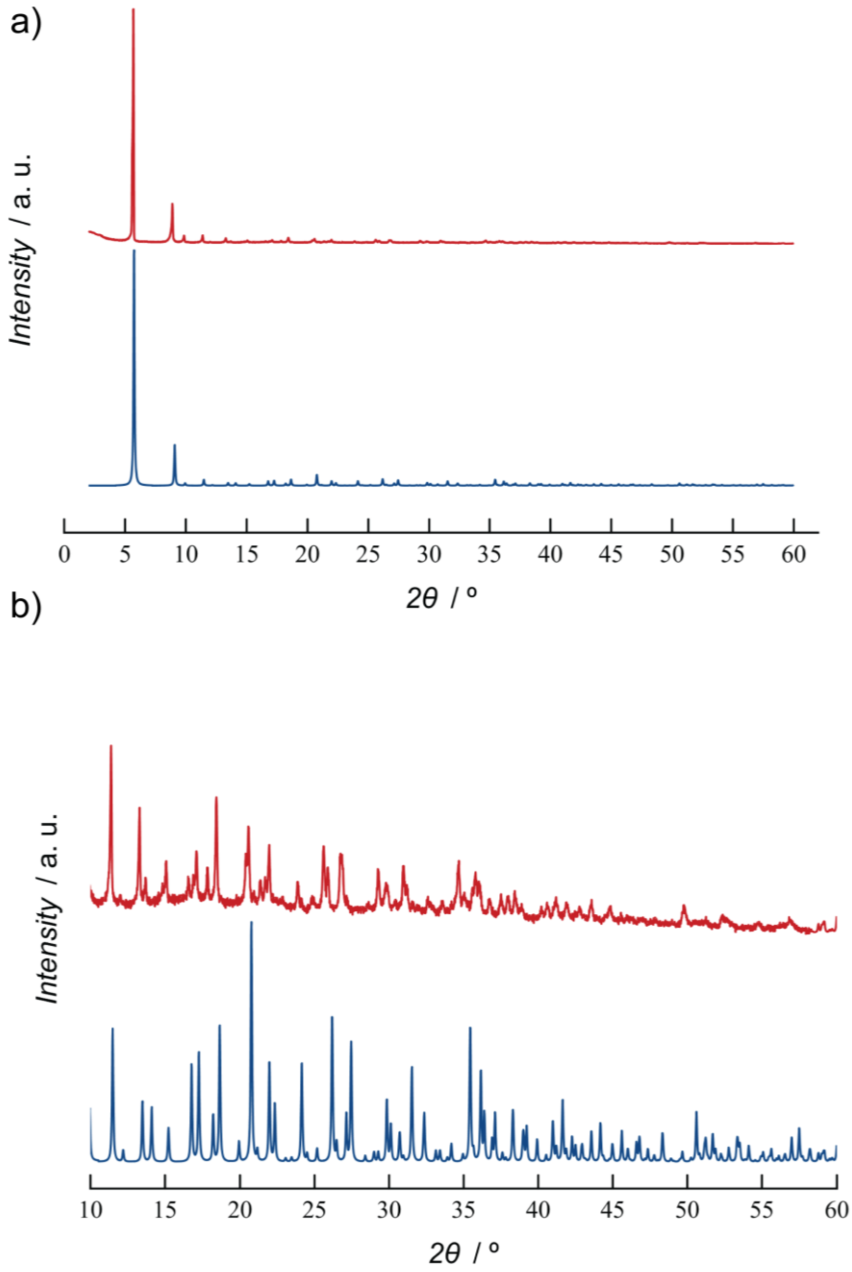

Figure S8. Theoretical (blue) and experimental (red) PXRD patterns of MTV-bioMOF **2** in the 2.0–60.0° (a) and 7.0–60.0° (b) 2θ range measured at room temperature.

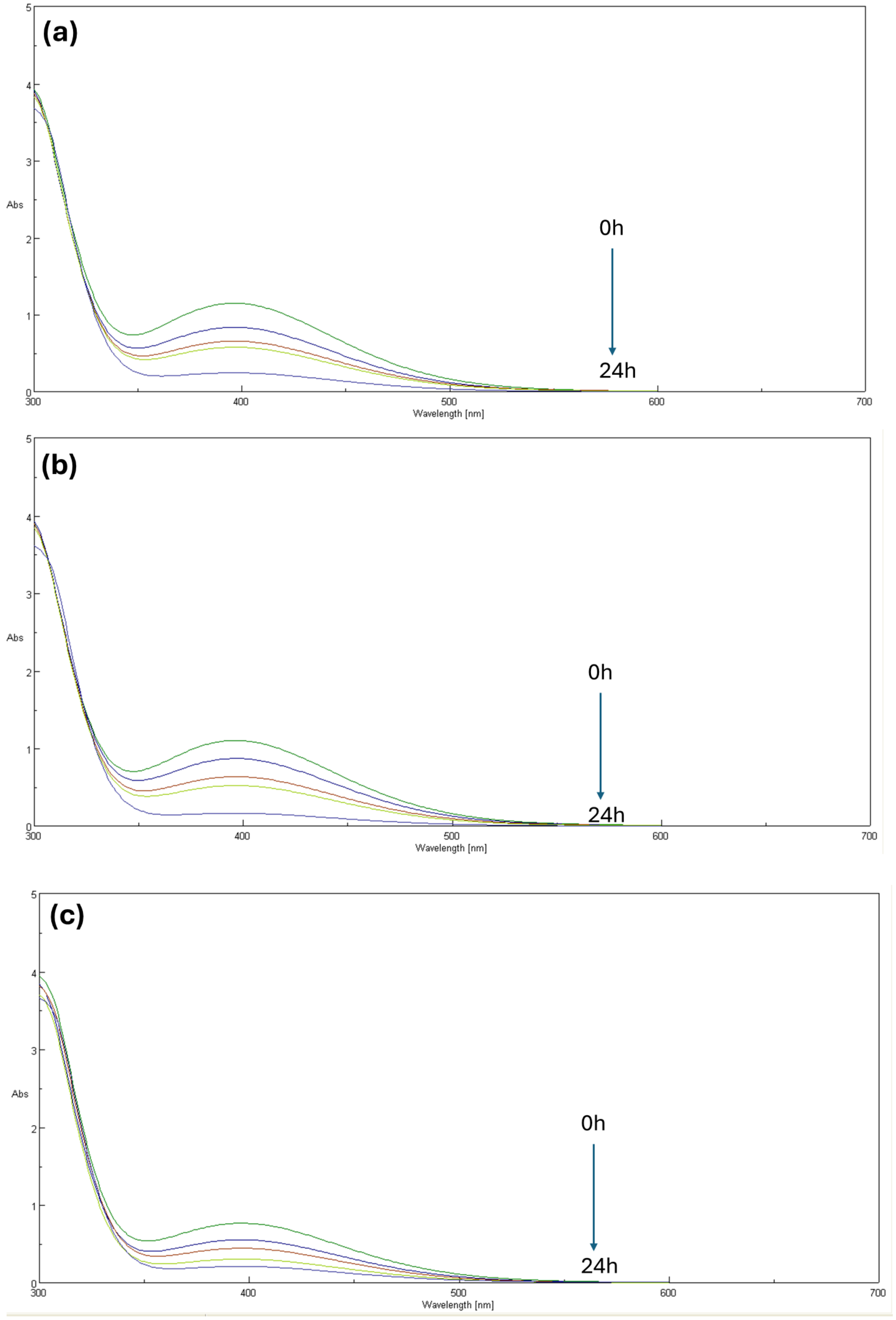

Figure S9. Reusability data for the catalase-like performance for MTV-bioMOF **2 s**olid in the decomposition of H_2O_2 at 37 ºC for run 1(a), run 2 (b) and run 3(c). UV-Visible spectra were measured at 0, 1, 3, 5 and 24h time intervals

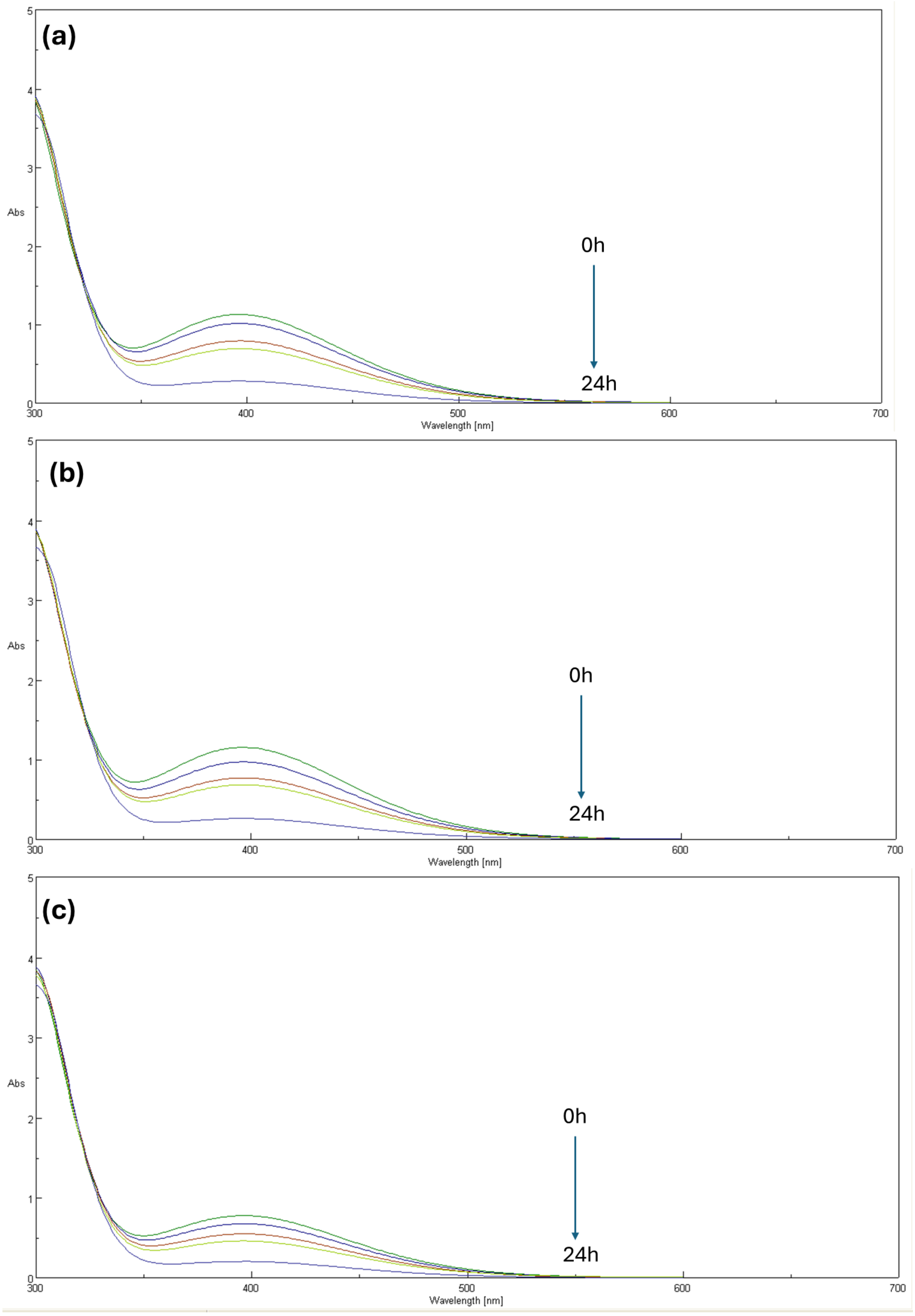

Figure S10. Reusability data for the catalase-like performance for bioMOF **1 s**olid in the decomposition of H_2O_2 at 37 ºC for run 1(a), run 2 (b) and run 3(c). UV-Visible spectra were measured at 0, 1, 3, 5 and 24h time intervals

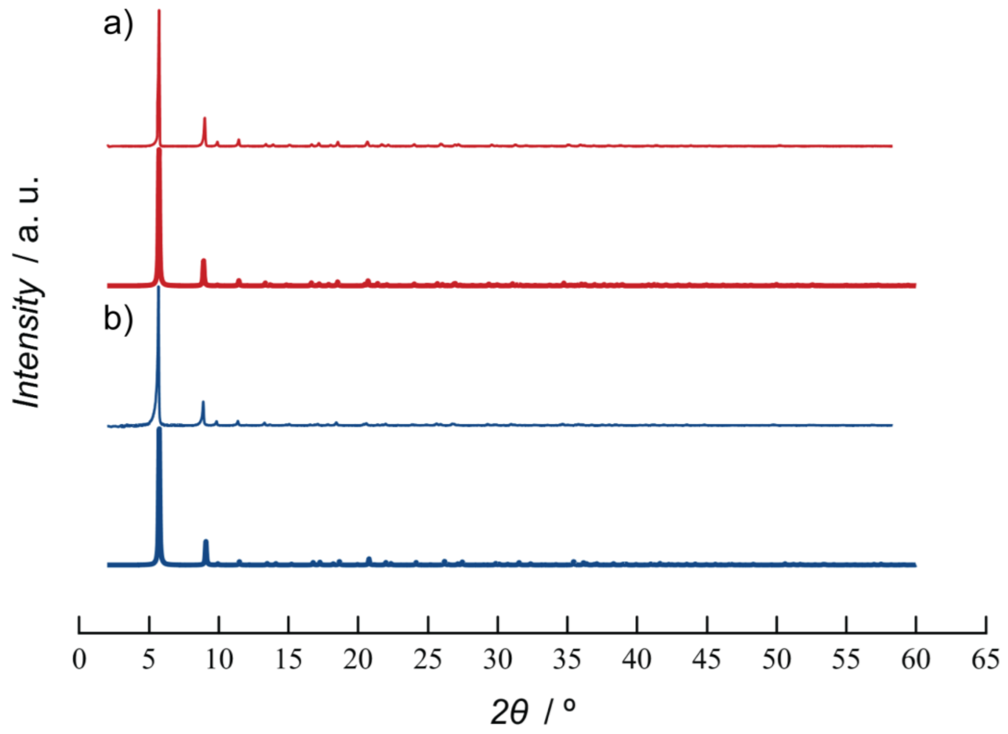

Figure S11. Theoretical (bold lines) and experimental (solid lines) PXRD patterns of MTV-bioMOF **1** (a, red) and 2 (b, blue) in the 2.0–60.0° 2θ range measured at room temperature after catalytic experiments.

References

(1) M. Mon, J. Ferrando-Soria, M. Verdaguer, C. Train, C. Paillard, B. Dkhil, C. Versace, R. Bruno, D. Armentano and E. Pardo, *J. Am. Chem. Soc.*, 2017, **139**, 8098–8101.

(2) M. Mon, R. Bruno, E. Tiburcio, P.-E. Casteran, J. Ferrando-Soria, D. Armentano and E. Pardo, *Chem. Eur. J.*, 2018, **24**, 17712–17718.

(3) (a) Evans, P. Sclaing and assessment of data quality. *Acta Cryst. D* **62**, 72–82 (2006). (b) Evans, P. R., Murshudov, G. N. How good are my data and what is the resolution?. *Acta Cryst. D* **69**, 1204–1214 (2013). (c) Winn, M. D. *et al*. Overview of the *CCP4* suite and current developments. *Acta Cryst. D* **67**, 235–242 (2011). (d) Winter, G. *xia2*: and expert system for macromolecular crystallography data reduction. *J. Appl. Cryst.* **43**, 186–190 (2010). (e) Winter, G. *et al*. *DIALS*: implementation and evaluation of a new integration package *Acta Cryst*. **2018**, D74, 85-97

(4) (a) Sheldrick, G. M. Crystal structure refinement with SHELXL. *Acta Cryst. C* **71**, 3–8 (2015). (b) Sheldrick, G. M. A short history of SHELX. *Acta Cryst. A* **64**, 112–122 (2008). (c) SHELXTL-2013/4, Bruker Analytical X-ray Instruments, Madison, WI, 2013.

(5) (a) Spek, A. L. *PLATON* SQUEEZE: a tool for the calculation of the disordered solvent contribution to the calculated structure factors. *Acta Crystallogr. Sect. C-Struct. Chem*. **71**, 9–18 (2015). (b) Spek, A. L. Structure validation in chemical crystallography. *Acta Crystallogr. Sect. D, Biol. Crystallogr.* **65**, 148–155 (2009).

(6) Farrugia, L. J. *WinGX* suite for small-molecule single-crystal crystallography. *J. Appl. Crystallogr.* **32**, 837–838 (1999).

(7) D. Palmer, CRYSTAL MAKER, Cambridge University Technical Services, C. No Title, 1996.

(8) R.M. Sellers. Spectrophotometric determination of hydrogen peroxide using potassium titanium(IV) oxalate. Analyst, 105 (1980), pp. 950-954).
